# Influence of Kombucha Fermentation on Antioxidant and Antimicrobial Activity of Monofloral Rapeseed Bee-Collected Pollen

**DOI:** 10.3390/antiox14060752

**Published:** 2025-06-18

**Authors:** Aleksandar Ž. Kostić, Aleksandra Sknepnek, Danijel D. Milinčić, Uroš Gašić, Sofija Kilibarda, Mirjana B. Pešić

**Affiliations:** 1 Chair of Chemistry and Biochemistry, Faculty of Agriculture, University of Belgrade, Nemanjina 6, 11080 Belgrade, Serbia; danijel.milincic@agrif.bg.ac.rs (D.D.M.); mpesic@ag.rif.bg.ac.rs (M.B.P.); 2 Chair of Industrial Microbiology, Faculty of Agriculture, University of Belgrade, Nemanjina 6, 11080 Belgrade, Serbia; alekavram@gmail.com; 3 Institute for Biological Research “Siniša Stanković”, National Institute of Serbia, University of Belgrade, 11000 Belgrade, Serbia; uros.gasic@ibiss.bc.ac.rs; 4 Department for Field and Vegetable Crops, Faculty of Agriculture, University of Belgrade, Nemanjina 6, 11080 Belgrade, Serbia; sofija.kilibarda@agrif.bg.ac.rs

**Keywords:** *Brassica napus* L. pollen, kombucha, phenolics, phenylamides, UHPLC Q-ToF-MS, biological activity

## Abstract

Bee-collected pollen (BCP) can serve as an excellent enhancer of functional food bioactivity, particularly when it is fermented. The aim of this study was to prepare a novel kombucha-based beverage (KPE) enriched with fermented monofloral rapeseed (*Brassica napus* L.) BCP. To characterize the obtained samples, a proximate phytochemical composition analysis (including total phenolic and flavonoid content) was performed, as well as a detailed untargeted UHPLC-Q-ToF-MS profiling of phenolics and phenylamides. To biologically characterize KPE, antioxidant and antimicrobial activities were monitored. The total phenolic and flavonoid content, enhanced by the addition of BCP to the kombucha green tea beverage, was dose-dependent. The control sample showed a strong predominance of flavan-3-ols, distinguishing it from the KPE samples, where flavonol predominance and an increased content of phenolic acids were observed. Notably, the most significant markers of BCP were phenylamides, which were completely absent in the control. Although antioxidant activity was proximately highest in the control sample, KPE samples exhibited significantly improved antimicrobial activity.

## 1. Introduction

Different foodstuffs can serve as sources of nutrients and bioactive compounds in the human diet. This is particularly true for plant-based food [1,2,3], and it is difficult to find a food that provides a better source of these nutrients than pollen. Pollen contains a balanced amount of sugars, proteins, and lipids, as well as an entire palette of various bioactive compounds, including phenolics, carotenoids, vitamins, sterols, alkaloids, etc. [4,5,6]. These properties make it an excellent functional food supplement and an “almost perfect food” [7,8]. Additionally, bee-collected pollen (BCP) has been recognized as an excellent source of pharmaceuticals [9] with notable potential for preventing various inflammatory processes, particularly neuroinflammation, as well as managing metabolic disorders such as obesity, diabetes, and certain cardiovascular disorders [6,9]. However, due to the presence of two resilient membranes (intended to protect sensitive pollen grains from unfavorable environmental factors), along with a non-polar layer called sporopollenin, the bioaccessibility and bioavailability of pollen’s nutrients and phytochemicals are often limited [10]. Recent research has confirmed that grinding BCP grains (a physical method) increases the bioaccessibility of various nutrients in camellia and lotus BCP, as measured after in vitro digestion of the samples [11].

The fermentation process is widely recognized as an enhancer of food quality. Through this process, new probiotics can be identified and incorporated into the diet, providing positive effects on human health [12]. Studies have shown that the nutritional quality of several vegetables improves after fermentation [13]. Among nutrients, proteins are particularly susceptible to fermentation, transforming into more desirable bioactive peptides with enhanced biological activity [14]. Interestingly, fermentation also reduces the content of antinutrients, including tannins, phytates, and certain proteins [13,14]. Given the various ways to improve the nutritional quality and bioactivity of BCP through different physical, chemical, or biological methods, fermentation is the most logical choice, as it naturally occurs in the hive after bees collect pollen. Through fermentation, bees transform BCP into a product known as bee bread (BB), or perga. Due to their shared botanical origin, BCP and BB have similar chemical compositions. However, fermentation is the primary factor driving the observed differences between these two bee products, especially regarding their free amino acids, free fatty acids, minerals, and phenolic content [15,16]. Studies have documented that BB possesses improved biological activity (particularly antioxidant activity) [17]. The most recent research also confirms BB’s antimicrobial properties, with potential to prevent microbial adhesion to inert surfaces [18].

Kombucha is a well-known beverage produced through the fermentation of black or green tea (*Camelia sinensis* L.), although other types of tea can also serve as substrates [19]. Kombucha is slightly acidic and carbonated, which contributes to its popularity among consumers. It can be made as a low-alcohol beverage (similar to sparkling wine) or as a non-alcoholic drink (with less than 0.5% (*v*/*v*)) [19]. Kombucha offers several benefits for consumers, including the presence of different probiotic microbes (such as acetic acid bacteria), amino acids, phenolics from tea, sugars, organic acids, ethanol, water soluble vitamins, etc. [19]. There have been efforts to enhance the quality of BCP through fermentation processes using lactic acid bacteria (LAB) strains on an industrial pilot-scale level [20] including the application of kombucha [21,22]. However, data on kombucha fermentation of monofloral BCP are still lacking, including a detailed profile of bioactive compounds and the biological activity of the resulting kombucha pollen-enriched (KPE) beverage. The importance of using monofloral BCP lies in ensuring a relatively consistent chemical composition. Therefore, the aim of this research was to prepare a KPE beverage by fermenting monofloral rapeseed (*Brassica napus* L.) BCP. The selected samples (control and samples with the highest BCP addition) were subjected to detailed untargeted UHPLC Q-ToF-MS analysis to obtain a precise profile of phenolics and phenylamides, the most important bioactive constituents of BCP. In addition, the antioxidant properties and potential antimicrobial activity of the KPE beverage were evaluated. The findings of this study could be useful to future researchers in developing a novel functional beverage with enhanced bioactivity, which could be popular among consumers who strive toward a modern but healthier lifestyle.

## 2. Materials and Methods

### 2.1. List of Reagents

For kombucha fermentation and microbial analysis, the sucrose used was provided by Sunoko d.o.o. (Kovačica, Serbia) and the green tea (*Camellia sinensis* L.) substrate was obtained from Welton, Jadar-pak d.o.o., Osečina (Serbia). For HPLC analysis, methanol and acetonitrile were purchased from Carlo Erba (Val-de-Reuil, France). The sodium hydroxide for all of the experiments was purchased from Zorka Šabac (Sabac, Serbia) as well as sodium carbonate. Folin–Cicolteu reagent was obtained from Sigma Aldrich (Steinheim, Germany). Sodium nitrite was purchased from Alkaloid, Skoplje (North Macedonia) while aluminum chloride hexahydrate was purchased from Sigma Aldrich (Steinheim, Germany). For antioxidant assays, the following chemicals were used: sulfuric acid (Zorka Šabac, Serbia), sodium phosphate dodecahydrate (Alkaloid, Skoplje, North Macedonia), sodium molybdate (Uni-chem, Belgrade, Serbia), ferric chloride hexahydrate (Sigma Aldrich, Steinheim, Germany), sodium dihydrogenphosphate and sodium hydrogenphosphate (Merck, Darmstadt, Germany), trichloroacetic acid (Merck, Darmstadt, Germany), cupric chloride dehydrate (Merck, Darmstadt, Germany), neocuproine (Sigma Aldrich, Steinheim, Germany), ammonium acetate (Merck, Darmstadt, Germany), DPPH (2,2-Diphenyl-1-picrylhydrazyl) radical (Sigma Aldrich, Steinheim, Germany), ABTS (2,2′-azino-bis(3-ethylbenzothiazoline-6-sulfonic acid)) radical cation (Sigma Aldrich, Steinheim, Germany), and methanol (Zorka Šabac, Serbia). For all experiments, mili-Q water was obtained with Smart-DUV apparatus (Amtast USA Inc., Lakeland, FL, USA). All reagents were of analytical grade except for the chemicals used for HPLC analysis, which were of HPLC purity grade.

### 2.2. Bee-Collected Pollen Sample Collection

**Figure 1:**
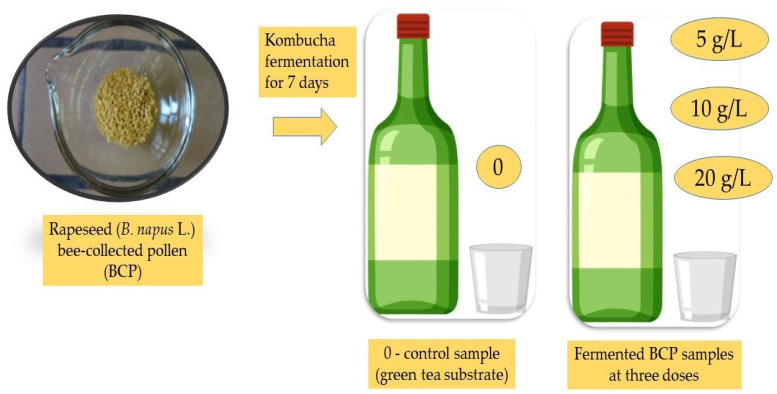
Appearance of rapeseed monofloral bee-collected pollen (BCP) and illustration of kombucha-fermented BCP preparation.

The BCP sample was obtained from a rapeseed plantation near Belgrade, courtesy of Professor N. Nedić. BCP was collected using pollen traps placed on the hive entrance. Pollen was collected over several days when the most intensive rapeseed (*Brassica napus* L.) was blossoming. Each day, after they were gathered from the traps, the grains were preliminary separated by shape and color and stored for preservation. At the end of the collection process, one representative sample was prepared and used for further palynological and chemical analyses. Its uniformity was confirmed (Figure 1) through color analysis, which showed a consistent light-yellow color. This was followed by palynological analysis, which verified that more than 99% of the pollen grains were from *B. napus* L., confirming the sample’s monofloral nature, with over 80% of pollen grains originating from rapeseed plants [23]. The sample was then transferred to the chemical laboratory at the Faculty of Agriculture, where it was carefully dried at a mild temperature (~40 °C) to achieve a desirable humidity level (~10%) to prevent spoilage. The sample was then vacuum-sealed and stored at 4 °C until the beverage preparation and further analyses.

### 2.3. Pollen-Enriched Kombucha Sample Preparation

To prepare enriched and non-enriched kombucha samples, 70 g/L of sucrose was added to water and heated to boiling. Then, 3 g/L of green tea (*Camellia sinensis* L.) was added and allowed to infuse for 15 min. The prepared tea (180 mL) was transferred to sterile 0.5 L glass bottles, which were sealed with cotton plugs, and cooled to room temperature. Enrichment with BCP was performed by mixing in various concentrations of pollen—5 g/L, 10 g/L, and 20 g/L. The enriched and non-enriched teas were inoculated with 20 mL of actively fermenting kombucha beverage. At the same time, tea samples enriched with BCP but not inoculated with fermenting kombucha were prepared for spontaneous fermentation. All samples were prepared in three replicates. The fermentation of all the samples was carried out at 25 ± 2 °C for 7 days. Spontaneously fermented enriched samples containing 5 g/L, 10 g/L, and 20 g/L BCP were labelled 1, 2, and 3, respectively, while their kombucha-fermented counterparts were labelled K1, K2, and K3. Kombucha-fermented, non-enriched green tea was designated to be sample 0. The titratable acidity of the samples was determined using 0.1 M NaOH with phenolphthalein as an indicator and expressed as grams of acetic acid per liter. The cellulose pellicles that formed were removed from the surface, and the samples were filtered and stored in the freezer until further analysis. A summary of KPE preparation is presented in Figure 1.

### 2.4. Proximate Phytochemical Characterization and UHPLC Q-ToF-MS Analysis

To broadly describe the phytochemical composition of the samples, the total phenolic content (TPC) and total flavonoid content (TFC) were determined in accordance with the literature [24]. The results were expressed in mg/100 mL. Following this, three representative samples were selected and subjected to solid phase extraction (SPE) pretreatment for UHPLC Q-ToF-MS analysis. These samples included the control sample (in order to determine the influence of green tea substrate on the phytochemical profile) and two samples with the highest BCP content (20 g/L), with and without kombucha fermentation, to try to identify and quantify all possible phytochemicals originating from BCP. The control and KBE samples (10 mL) were passed through preconditioned SPE cartridges (CLEAN-UPR, C18 Extraction columns, Unendcapped-PKG50, UCT, Bristol, UK) to concentrate bioactive compounds and remove residual sugars and other impurities. The SPE cartridges were conditioned with 5 mL of acidified methanol and 5 mL of milliQ water, while adsorbed phenolics and phenylamides were eluted with acidified methanol (methanol containing 0.1% HCl). The samples were then filtered through 0.22 µm syringe filters and analyzed using UHPLC Q-ToF-MS. The kombucha samples were analyzed with an Agilent 1290 Infinity ultra-high-performance liquid chromatography (UHPLC) system coupled with a quadrupole time-of-flight mass spectrometer (6530C Q-ToF-MS, Santa Clara, CA, USA). All parameters and conditions for UHPLC Q-ToF-MS profiling were as previously reported [25]. Briefly, a Zorbax C18 column (2.1 × 50 mm, 1.8 µm) was used for chromatographic separation. Mobile phases comprised the following: (A) ultrapure water+0.1% HCOOH and (B) 98% acetonitrile+0.1% HCOOH) (MS grade). The flow rate was set at 0.3 mL/min, and the injection volume was 5 µL. The ESI operation parameters were the same as previously reported by Kostić et al. [25]. These included a nebulizer pressure of 45 psi, a drying gas temperature of 225 °C, a flow rate of 8 L/min, a sheath gas temperature of 300 °C, a sheath gas flow rate of 10 L/min, a capillary voltage of 2500 V, a fragmentor energy value of 175 V, a skimmer voltage of 65 V, and an octopole RF peak at 750 V. Phenolic compounds were detected in negative ionization mode, while the analysis of phenylamides was performed in positive ionization mode, using auto MS/MS acquisition (*m*/*z* = 50–1700, scan rate 1 spectra/s). All bioactive compounds were identified based on their monoisotopic mass, MS fragmentation, and literature data [26,27,28,29,30,31,32].

Phenolic compounds were quantified, and the results were expressed as µg/100 mL of the obtained beverages. Due to the lack of specific standards, the content of some phenolic derivatives was expressed in equivalents of the structurally closest (available) standard (gallic acid for phenolic acids and derivatives; epicatechin for flavan-3-ols and derivatives; quercetin for flavanol aglycones and glycosides; and naringenin for flavanones). Quantification parameters (equation parameters, coefficient of determination, and limit of quantification) for the applied standards are given in Table S1. The relative content of individual phenylamides (%) was calculated as the ratio of the area of each individual phenylamide to the total identified phenylamides.

### 2.5. Determination of Antioxidant Properties

To fully understand the bioactivity of the obtained samples, six different standard antioxidant assays were employed: in vitro phosphomolybdenum total antioxidant capacity (TAC), ferric reducing power (FRP), cupric reducing antioxidant capacity (CUPRAC), 2,2-diphenyl-1-picrylhydrazyl (DPPH^•^) radical quenching ability, (2,2-azino-bis-3-ethylbenzothiazoline-6-sulphonic acid) radical cation (ABTS^•+^) quenching ability, and Fe^2+^ chelating ability. Detailed descriptions of the experimental procedures for TAC, FRP, CUPRAC, DPPH^•^, and ABTS^•+^ assays are available in prior research [24].

### 2.6. Determination of Antimicrobial Activity

To assess the antimicrobial properties, pollen-enriched kombucha samples were prepared and labelled as described in Section 2.2. To remove the antimicrobial influence of the synthesized acids, neutralized samples were also prepared by adding 0.1 M NaOH to achieve a pH of 7.0, and the samples were labelled as 0N, 1N, 2N, 3N, K1N, K2N, and K3N. Prior to analysis, all samples were filtered through 0.22 µm filters (Sartorius, Goettingen, Germany). For antimicrobial testing, four Gram-positive bacteria (*Bacillus spizizenii* ATCC 6633, *Staphylococcus aureus* ATCC 25923, *Staphylococcus aureus* clinical isolate, *Listeria monocytogenes* ATCC 19111), five Gram-negative bacteria (*Pseudomonas aeruginosa* ATCC 35302, *Escherichia coli* ATCC 25922, *Salmonella enterica* subsp. *enterica* serovar Enteritidis ATCC 13073, *Klebsiella aerogenes* ATCC 13048, *Acinetobacter baumannii* ATCC 19606), and one pathogenic yeast (*Candida albicans* ATCC 10231) were used. The antimicrobial activity of the samples was determined using a microdilution assay as previously described [33]. The results were expressed as minimal inhibitory concentration (MIC), minimal bactericidal concentration (MBC), or minimal fungicidal concentration (MFC) in % of the sample content, which ranged from 50% to 0.39%. Antibiotic penicillin and antimycotic nystatin were used to analyse the susceptibility of tested strains in concentrations that ranged from 20 to 0.05 mg/mL.

### 2.7. Statistical Analysis

Statistical analysis was conducted using R Studio 4.3.1 software, employing analysis of variance (ANOVA) and Tukey’s post hoc test to determine the statistically significant differences (*p* < 0.05).

## 3. Results and Discussion

### 3.1. Proximate Phytochemical Composition of Prepared Samples

Previous studies confirm that extended kombucha fermentation (over 10 days) may diminish the beverage’s refreshing and pleasant sensory properties, resulting in a vinegar-like taste [34]. Therefore, a 7-day fermentation period was selected for this study. The results for proximate phytochemical composition, obtained through total phenolic (TPC) and flavonoid (TFC) content determination, are presented in Table 1.

Pollen addition clearly enriched the prepared samples with additional bioactive compounds belonging to both groups. The highest TPC values were observed in the samples with 20 g of added pollen, regardless of kombucha fermentation, the results of which were not statistically different. However, for TFC, kombucha fermentation with the highest pollen content (20 g) resulted in the highest measured TFC. This outcome is expected, as flavonoids and their derivatives are known to be the most important class of phenolic compounds in BCP [35,36,37]. Similarly, authors from Romania observed increased TPC content in kombucha after pollen addition [21].

### 3.2. The Obtained UHPLC Q-ToF-MS Profile of Selected Samples

A detailed comparative UHPLC-Q-ToF-MS analysis was conducted on the control sample (kombucha-fermented green tea) alongside BCP samples, both without and with kombucha fermentation (samples 3 and K3, respectively) to determine specific biomarkers. To the best of the authors’ knowledge, this is the first report with detailed untargeted phenolics/phenylamides analysis of KPE samples available in the literature. Based on the results presented in Table 2, the prepared beverages displayed distinct phytochemical profiles.

The control sample (0), consisting of green tea fermented with kombucha, had a significantly different profile compared to the samples with 20 g of pollen addition (3 and K3). In total, sample 0 contained 15 flavonols and derivatives, 12 phenolic acids and derivatives, eleven flavan-3-ols and derivatives, and two flavones. However, a strong quantitative predominance of flavan-3-ols/derivatives was observed (14,842 µg/100 mL) compared to flavonols/derivatives (660 µg/100 mL), flavones (364 µg/100 mL), and phenolic acids/derivatives (26.1 µg/100 mL). The profile observed for sample 0 was expected due to the presence of green tea, in which flavan-3-ols/derivatives are known to comprise the main phenolic subclass [44]. Luteolin (flavone, 364 µg/100 mL) was quantified only in sample 0, suggesting its relation to the tea substrate. Among other, more specific compounds identified and/or quantified only in sample 0, galloyl-HHDP-hexose (26 µg/100 mL) was the only phenolic acid derivative. Additionally, three flavan-3-ols were determined as specific to sample 0: epiafzelechin-3-*O*-gallate (108 µg/100 mL), epigallocatechin-hexoside (74 µg/100 mL), and epigallocatechin-epigllocatechin 3-*O*-gallate (199 µg/100 mL). Interestingly, epiafzelechin-3-*O*-gallate is a highly specific metabolite of the *Camelia sinensis* plant, i.e., green tea [45]. Among flavonol derivatives, only two tea-related compounds were detected: myricetin-3-*O*-hexoside (31 µg/100 mL) and quercetin 3-*O*-(2″-rhamnosyl-3″-pentosyl-6″-coumaroyl)-hexoside (39 µg/100 mL). Kaempferol-3-*O*-rhamnoside was also identified, but it was below the LOQ. In contrast, samples 3 and K3 were characterized by the presence of naringenin, a flavanone not found in sample 0, while eriodyctiol was present but at a level below the LOQ. Sample 3 showed a qualitative phenolic profile with 17 flavonols/derivatives, 13 phenolic acids/derivatives, and nine flavan-3-ols/derivatives. In sample K3, there were additional increases in flavonols/derivatives (18 compounds) and phenolic acids/derivatives (14 compounds), while flavan-3-ol diversity decreased to eight compounds. However, compared to the control, the quantitative profile was completely altered. While the total amount of flavan-3-ols was significantly reduced (~3300–4130 µg/100 mL), flavonols were quantified in samples 3 and K3 at 3070 and 3330 µg/100 mL, respectively. This confirmed that kombucha fermentation positively influenced the enriched profile and bioavailability of flavonols in the obtained beverages. The total amount of phenolic acids/derivatives was 199 µg/100 mL in sample 3 and 187 µg/100 mL in sample K3. Previous studies indicate that the amount of phenolic acid in fermented BCP fluctuates depending on specific compounds. For example, in one study, caffeic, ferulic acid, and ellagic acid contents increased, while chlorogenic acid content decreased after fermentation [46]. Regarding specific biomarkers, a higher diversity was observed originating from pollen compared to the control. Three phenolic acids were identified/quantified: cinnamic acid (45.3 µg/100 mL), dihydroxybenzoic acid (26.5–58 µg/100 mL), and feruloylquinic acid (below LOQ). Among flavonols/derivatives, as many as seven compounds were identified/quantified, including, among others, quercetin 3-*O*-(2″-hexosyl)-hexoside (225–293 µg/100 mL), kaempferol 3-*O*-(2″-hexosyl)-hexoside (168–181 µg/100 mL), kaempferol 3-*O*-(2″-rhamnosyl-6″-hexosyl)-hexoside (142 µg/100 mL in both samples), kaempferol 3-*O*-(2″-*O*-pentosyl)-hexoside (below LOQ), and quercetin 3-*O*-(2″-hexosyl)hexoside-7-*O*-hexoside (below LOQ). Notably, kaempferol 3-*O*-(6″-hexosyl)-hexoside-7-*O*-hexoside (47 µg/100 mL) was quantified only in the kombucha-fermented sample, possibly due to kombucha activity.

**Figure 2:**
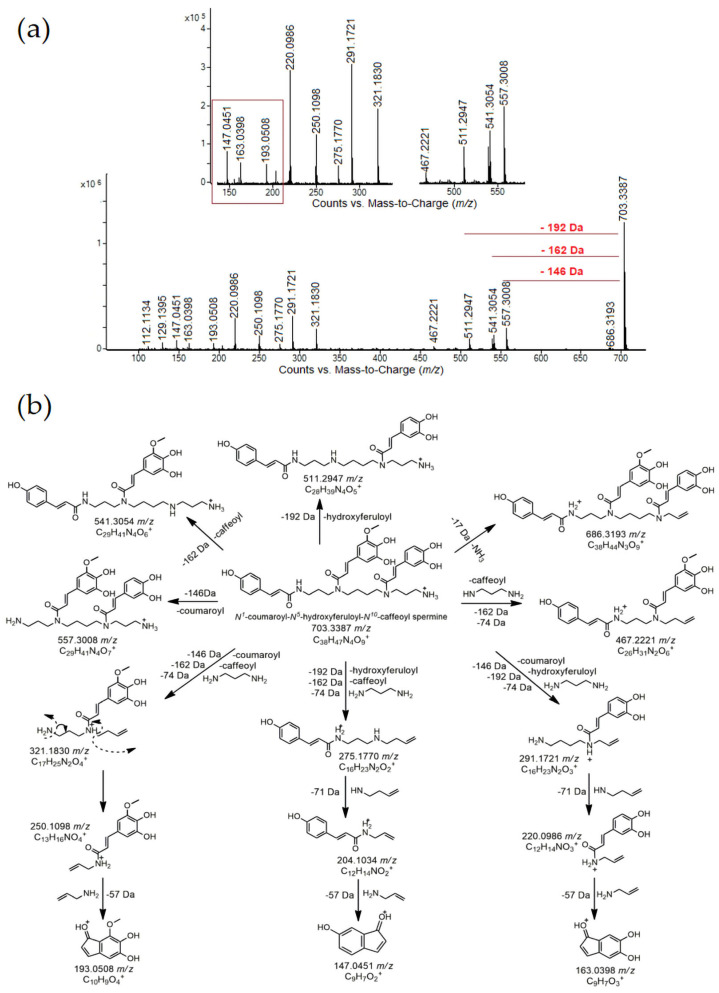
(**a**) Characteristic MS/MS fragmentation pattern (collision-induced dissociation (CID) mass spectra) and (**b**) proposed fragmentation pathway corresponding to *N^1^*-coumaroyl-*N^5^*-hydroxyferuloyl-*N^10^*-caffeoyl spermine (703 *m*/*z*) (Agilent, Q-ToF, ESI(+), CE = 30 eV).

However, more informative and conclusive data were obtained through UHPLC monitoring of the phenylamide profiles (Table 3). This group of bioactive compounds was completely absent in the control sample, confirming its exclusive connection to BCP. Phenylamides were overlooked in BCP for decades, likely due to the absence of appropriate standards and highly sophisticated analytical techniques. However, in recent years, several reports [25,47,48] have documented their presence in BCP, with profiles strongly influenced by the botanical origin of the pollen. Even more interestingly, previous authors have reported that phenylamides are the most abundant group of bioactive compounds in BCP, compared to flavonoids/derivatives [47]. To the best of our knowledge, this study is the first to identify and characterize phenylamides derived from rapeseed BCP. In the current study, 13 derivatives from this group of compounds were identified. Based on their polyamine core, these phenylamides can be classified into three groups: (1) spermine derivatives (eight compounds), (2) spermidine derivatives (four compounds), and (3) a putrescine derivate (one compound). The structures and names of all identified phenylamides, along with the most likely positions of phenolic acid moieties on the spermidine/spermine core, were proposed based on typical fragments (Table 3). Individual or combined coumaroyl, caffeoyl, feruloyl, and hydroxyferuloyl derivatives of spermidine and spermine were confirmed primarily in KPE beverages, with the coumaroyl moiety distinctly dominant. All identified spermidine derivatives contained one, two, or three coumaroyl moieties (compounds **2**–**4**, Table 3). Key fragments for the identification of these compounds were observed at *m*/*z* 147 (coumaroyl moiety), *m*/*z* 204 (coumaroyl propenamide), *m*/*z* 292 (coumaroyl spermidine)→275 (-NH_3_), and *m*/*z* 438 (di-coumaroyl spermidine)→421 (-NH_3_). These coumaroyl spermidine derivatives were previously found in various monofloral and polyfloral BCP samples [25,32,49,50]. Fragments observed at *m*/*z* 177, *m*/*z* 234, and *m*/*z* 322 indicate the presence of a feruloyl moiety in the spermidine structure (compound **4**, Table 3). In contrast to previous studies that mostly identified spermidine derivatives in pollen, this study detected eight specific spermine derivatives which could serve as potential chemotaxonomic markers for rapeseed BCP. All identified derivatives contained coumaroyl, caffeoyl, feruloyl, or hydroxyferuloyl moieties in various positions on the spermine core. The presence of tricoumaroyl spermine in pollen has been confirmed in several studies [25,32], while dicoumaroyl feruloyl spermine and dicoumaroyl caffeoyl spermine were identified and reported by [32] in analyses of eight monofloral BCP samples from China. Of particular interest are spermine derivatives containing a hydroxyferuloyl residue, indicated by fragments at *m*/*z* 193 (hydroxyferuloyl moiety), *m*/*z* 250 (hydroxyferuloyl propenamide), and *m*/*z* 321. Compound **11** (Table 3) was identified as *N^1^*-coumaroyl-*N^5^*-hydroxyferuloyl-*N^10^*-caffeoyl spermine, with its proposed fragmentation pathway shown in Figure 2.

The structures of the other spermine derivatives were proposed based on typical fragments, as detailed in Table 3. Moreover, tentatively identified compounds and their fragments were additionally confirmed and supported by available literature, related to previously publish mass spectrometry data [38,51,52,53,54,55]. However, the proposed phenylamide structures should be confirmed in future studies using NMR or other analytical techniques.

Due to the lack of appropriate standards, the relative content of individual phenylamides was calculated to indicate their presence/dominance in the samples. In both samples containing pollen, the phenylamide identified as *N^1^,N^5^*-dicoumaroyl spermidine was the predominant compound, accounting for 55–56% of the total phenylamides detected. This was followed by compounds recognized as *N^1^,N^5^*-di-coumaroyl-*N^10^*-hydroxyferuloyl spermine (16–17%) and *N^1^*-coumaroyl-*N^5^*-hydroxyferuloyl-*N^10^*-feruloyl spermine (5–5.6%). These results indicate that coumaroyl derivatives were the most predominant, originating from the initial BCP obtained from rapeseed. Since there are no data available on pollen-enriched beverages for comparison, the phenylamides’ profile will be matched to previously published monofloral BCP samples. For instance, coconut BCP was characterized by the presence of tricoumaroyl spermidine [36] unlike rapeseed BCP, where this compound was only present in trace amounts (0.01–0.02%). Additionally, *Castanea* sp. BCP from Spain contained tricoumaroyl spermidine and a qualitative predominance of caffeoyl spermidine derivatives [56], though quantification was not performed. Corn poppy monofloral BCP from Serbia showed significantly higher diversity in these compounds, with 27 different derivatives and a strong predominance of coumaroyl spermine derivatives [25]. Research from Portugal also reported a tetracoumaroyl spermine isomer (3.3 mg/g) as the most predominant bioactive compound in BCP, primarily from *Plantago* sp. and *Crepis capillaris* plants [47]. The provided data and observed differences highlight the importance of phenylamides as carriers of BCP bioactivity and underscore their potential as a strong tool for BCP chemotaxonomy.

### 3.3. Antioxidant Properties of Obtained Samples

Analyzing the antioxidant properties of a foodstuff is an important means of gaining insights into its biological activity. Consequently, the KPE sample, the pollen-enriched sample without kombucha, and the control sample were analyzed using five standard antioxidant assays, and the results are presented in Table 1. It can be observed that for all applied assays, except the in vitro phosphomolybdenum total antioxidant capacity (TAC), the control sample exhibited the highest activities. In all pollen-enriched samples, there were significant decreases in the obtained values, which is likely linked to the reduction in flavan-3-ols content in all pollen-enriched samples, discussed in detail in the previous section. Indeed, flavan-3-ols are exceptional antioxidants [57]. Although pollen-enriched samples contained a significantly higher content of flavonols and phenolic acids, it has been reported that the antioxidant activity of different phenolic subclasses decreases in the following order: procyanidin dimers (derivatives of catechins) < flavanols < flavonols < hydroxycinnamic acid derivatives < simple phenolic acids [58]. It can be speculated that the decrease in flavan-3-ol content may be due to increased microbial activity in pollen-enriched samples, resulting from the high nutrient availability in pollen. Flavan-3-ols from tea, for instance, are known to be metabolized by microbes in the human colon, transforming into several metabolites, including methyl, glucuronide, and sulphate derivatives [59]. For TAC values, the highest antioxidant capacity was observed in the sample without kombucha fermentation (31) containing 5 g/L of pollen with 212.7 mg/100 mL AAE. It is possible that the high sugar content in BCP (significantly higher than in green tea) contributed to the higher or comparable antioxidant values in all pollen-enriched samples relative to the control. Rapeseed BCP, in particular, contains sugars as the predominant nutrients, comprising more than 62% d.w. [60]. In pollen-enriched samples, kombucha fermentation was associated with increased antioxidant activity in all KPE samples across assays (FRP; CUPRAC, DPPH^•^, ABTS^•+^) compared to the samples without kombucha, except in TAC, as explained earlier. Interestingly, in all assays except TAC, the KPE beverage with 5 g/L of pollen exhibited the highest antioxidant activity, suggesting that this pollen concentration may be optimal for enhancing antioxidant capacity. With higher pollen content, potential interferences may arise due to the complexity of the food matrix, which includes various nutrients from pollen, phytochemicals from multiple classes, and microbes from the kombucha consortium. 

### 3.4. Antimicrobial Activity of Obtained Samples

Minimal inhibitory, minimal bactericidal, and minimal fungicidal concentrations were determined for the obtained samples of green tea kombucha, pollen-enriched green tea kombucha, and spontaneously fermented green tea with BCP. The results are presented in Table 4. Additionally, these values were measured for neutralized samples to exclude the influence of product acidity. The acidity levels in the fermented samples were found to be 0.45 g/L, 1.1 g/L, 1.6 g/L, 1.9 g/L, 2.2 g/L, 2.3 g/L, and 2.8 g/L for samples 0, 1, 2, 3, K1, K2, and K3, respectively.

The spontaneously fermented sample 1, with the lowest concentration of added pollen (5 g/L), exhibited antimicrobial and antifungal activity against all tested microorganisms, with inhibition values lower than those of microbicidal activity. The neutralized sample 1 (1N) retained inhibitory activity against all Gram-negative bacterial strains and against *L. monocytogenes* among the Gram-positive bacteria, though it did not show any microbicidal properties. Following kombucha fermentation of the pollen-enriched green tea (K1), the antimicrobial activity was partially improved compared to sample 1, especially regarding the MBC values. This improvement was mainly due to the higher acidity, as the neutralized K1N sample demonstrated better activity with a lower MIC value only against *P. aeruginosa*, but it showed weaker activity against *K. aerogenes* and *E. coli* compared to sample 1N. The addition of a medium concentration of pollen (10 mg/L) did not improve the antibacterial activity of the spontaneously fermented sample. The improved activity of sample 2 compared to sample 1 is attributed to its higher acidity, as shown by the equal activity of neutralized samples 1N and 2N, except in the case of *S. aureus* ATCC, where samples 1 and 1N exhibited better activity. The fermented kombucha beverage with 10 mg/L of added pollen (sample K2) expressed improved inhibitory activity against *A. baumannii*, better bactericidal activity against *S. aureus* ATCC 25293, and better fungicidal activity compared to sample 2. However, sample 2 showed better inhibitory activity against the clinical isolate of *S. aureus* and better bactericidal activity against *S.* Enteritidis. After neutralization, sample K2N showed better inhibitory activity against *A. baumannii* and both *S. aureus* species compared to all neutralized samples at 5 mg/L and 10 mg/L (1N, K1N, and 2N) and a lower MIC for *K. aerogenes* than K1N. The inhibitory effect of sample K2N on other species remained the same as for the K1N sample.

Sample 3, with the highest pollen concentration (20 mg/L) and spontaneous fermentation, showed lower MIC values against *K. aerogenes*, *A. baumannii,* and *S. aureus* ATCC 25293 compared to sample 2. Additionally, MBC values for *P. aeruginosa* and both *S. aureus* species, as well as MFC values for *C. albicans*, were lower.

However, sample 3 demonstrated weaker bactericidal activity against *S.* Enteritidis compared to sample 2. After neutralization, sample 3N showed better inhibitory activity than sample 2N against *P. aeruginosa*, *A. baumannii,* and both *S. aureus* strains, although bactericidal and fungicidal activities were absent. The kombucha beverage with 20 mg/L pollen (K3) showed improved inhibitory activity against *K. aerogenes*, both *S. aureus* species, and *B. subtilis* and better bactericidal activity against *S. aureus* and *L. monocytogenes* compared to sample K2.

None of the neutralized samples, whether spontaneously fermented or fermented with kombucha, exhibited bactericidal activity until the pollen concentration was raised to 20 mg/L and fermented by a kombucha consortium. Sample K3N showed bactericidal activity against *P. aeruginosa* at a concentration of 25% and against *K. aerogenes* at a concentration of 50%. In addition, K3N showed inhibitory activity against all bacteria, with the strongest effect observed against *B. subtilis*. Chemical analysis of sample K3 indicated the highest content of total phenolic compounds (Table 1). UHPLC-QToF-MS analysis (Table 2) further revealed that sample K3 contained higher amounts of cinnamic acid, gallic acid hexoside, and hydroxybenzoic acid isomer I compared to sample 3. Moreover, K3 had an increased total content of flavonol aglycones and glycosides, which likely contributed to its enhanced antimicrobial activity. Bioactive compounds in pollen, such as phenolic and flavonoid compounds, are known to be antimicrobial agents. Additionally, pollen-related bacterial strains such as *Streptomyces*, known as antibiotic-producing strains, are associated with the suppression of pathogenic microorganisms.

It is important to emphasize that all pollen-enriched samples showed better antimicrobial activity compared to green tea kombucha samples (0 and 0N). The only exception was with the clinical isolate of *S. aureus*, where only samples K2N, 3N, and K3N had the same effect as the neutralized green tea kombucha sample. *S.* Enteritidis was the most sensitive to both acidic and neutralized samples, with MIC values of 1.6% and 3.1%, respectively. Sample K3 also showed effectiveness against the clinical isolate of *S. aureus,* with an MIC of 1.6%. Generally, Gram-negative bacteria were more sensitive than Gram-positive, whereas antibiotic penicillin was primarily effective against Gram-positive bacterial species. This may indicate that the kombucha samples possessed bioactive compounds that act through mechanisms different from those of β-lactam antibiotics, with the potential to overcome the outer membrane of Gram-negative bacteria. As the neutralized samples did not affect *C. albicans*, it can be concluded that the antifungal activity was due only to the synthesized acids. These results show that the production of acids during the kombucha fermentation process is a suitable way to inhibit the growth of microorganisms and increase the shelf life and safety of a product by preventing spoilage and inhibiting pathogenic microorganisms [33].

## 4. Conclusions

Beverages with improved health functions are gaining popularity. In this study, the addition of fermented rapeseed bee-collected pollen (BCP) to a kombucha green tea-based beverage aimed to create a novel, healthier beverage. BCP was found to enhance the total phenolic content and increase the diversity of bioactive compounds, as identified and quantified using UHPLC-Q-ToF-MS analysis. Although the control sample (without BCP) exhibited the highest antioxidant activity in almost all assays (mostly due to the presence of flavan-3-ols and derivatives), KPE samples demonstrated superior antimicrobial activity compared to the control sample. Gram-positive bacteria were generally more susceptible to KPE’s antibacterial activity compared to Gram-negative strains. At the highest dose of BCP in the kombucha beverage, improved activity was observed against *K. aerogenes*, *S. aureus*, and *B. subtilis*, as well as enhanced bactericidal activity against *L. monocytogenes*. Further research should focus on the sensory analysis of KPE, as well as further investigation into potential anticancer and anti-inflammatory properties of the BCP-enriched kombucha beverage to support its potential health benefits.

## Figures and Tables

**Table 1:** Proximate phytochemical composition and antioxidant characterization of kombucha enriched with BCP, expressed as mg equivalents **/100 mL of sample. All results are expressed as mean value ± st. dev.

Sample	TPC * (GAE **)	TFC (QE)	TAC (AAE)	FRP (AAE)	CUPRAC (AAE)	DPPH^•^ (TE)	ABTS^•+^ (TE)
0	16.98 ± 0.11 ^e^	31.97 ± 0.14 ^a^	156.46 ± 0.59 ^c^	46.70 ± 0.13 ^a^	424.20 ± 16.85 ^a^	31.34 ± 0.89 ^a^	34.87 ± 0.51 ^a^
1	21.94 ± 0.17 ^d^	12.84 ± 0.93 ^c^	212.66 ± 0.37 ^a^	13.96 ± 0.16 ^d^	213.76 ± 4.06 ^c^	10.14 ± 0.10 ^b,c^	18.08 ± 0.71 ^d^
K1	23.45 ± 0.33 ^c^	11.99 ± 0.21 ^c^	188.42 ± 1.58 ^b^	15.85 ± 0.36 ^b^	272.35 ± 14.47 ^b^	10.69 ± 0.07 ^b^	22.16 ± 0.54 ^b^
2	25.31 ± 0.35 ^b^	12.70 ± 0.36 ^c^	180.70 ± 4.42 ^b^	11.10 ± 0.19 ^f^	196.52 ± 27.28 ^c^	9.81 ± 0.06 ^b,c^	16.66 ± 0.57 ^e^
K2	24.66 ± 0.21 ^b^	11.77 ± 1.29 ^c^	159.81 ± 3.78 ^c^	12.67 ± 0.36 ^e^	204.05 ± 12.82 ^c^	10.33 ± 0.01 ^b,c^	18.68 ± 0.37 ^c,d^
3	27.03 ± 0.59 ^a^	12.56 ± 0.07 ^c^	155.93 ± 1.34 ^c^	13.66 ± 0.37 ^d^	212.44 ± 5.03 ^c^	9.53 ± 0.01 ^c^	18.21 ± 0.03 ^d^
K3	26.46 ± 0.32 ^a^	14.77 ± 0.43 ^b^	114.43 ± 3.88 ^d^	14.88 ± 0.14 ^c^	212.44 ± 5.03 ^c^	9.99 ± 0.03 ^b,c^	19.56 ± 0.03 ^c^

0—control sample (contains only tea substrate); 1, 2, 3—samples fermented without kombucha enriched with 5, 10, and 20 g/L pollen, respectively; K1, K2, K3—samples fermented with kombucha enriched with 5, 10, and 20 g/L pollen, respectively; * TPC—total phenolic content; TFC—total flavonoid content; TAC—in vitro phosphomolybdenum total antioxidant capacity; FRP—ferric reducing power; CUPRAC—cupric reducing antioxidant capacity; DPPH^•^—2,2-Diphenyl-1-picrylhydrazyl radical quenching ability; ABTS^•+^—(2,2-azino-bis-3-ethylbenzothiazoline-6-sulphonic acid) radical cation quenching ability; ** GAE—gallic acid equivalents; QE—quercetin equivalents; AAE—ascorbic acid equivalents; TE—Trolox equivalents. Different lowercase letters (a–f) in the same column indicate significant differences among samples, as determined using analysis of variance (ANOVA) and Tukey’s post hoc test, with a significance level of *p* < 0.05.

**Table 2:** Identification and quantification (µg/100 mL of sample) of phenolic compounds and their derivatives in kombucha beverages with/without bee-collected pollen, using UHPLC-QToF-MS.

RT	Compound Name	Formula	Calculated Mass	*m*/*z* Exact Mass	mDa	Fragments (MS^2^)	Refs. *	Samples (µg/100 mL)		
								0	3	K3
Phenolic acids and derivatives										
5.73	**Hydroxybenzoic acid isomer I ^b^**	C_7_H_5_O_3_^−^	137.02390	137.02480	−0.90	/	[25,38]	<LOQ	<LOQ	25.73
8.83	**Hydroxybenzoic acid isomer II ^b^**	C_7_H_5_O_3_^−^	137.02390	137.02509	−1.19	**/**	[25,38]	<LOQ	<LOQ	<LOQ
7.75	**Cinnamic acid ^b^**	C_9_H_7_O_2_^−^	147.04460	147.04543	−0.83	101.0412(68), 103.05682(7), **117.03663(100)**, 118.03995(10), 119.05082(7)	[35,39]	-	46.58	74.58
3.44	**Dihydroxybenzoic acid ^b^**	C_7_H_5_O_4_^−^	153.01880	153.01970	−0.90	**108.02333(100)**, 109.03079(77)	[25,37]	-	45.29	<LOQ
1.68	**Gallic acid ^a^**	C_7_H_5_O_5_^−^	169.01370	169.01470	−1.00	107.01514(10), 108.02348(9), 123.00827(12), **124.01806(84)**, **125.02585(100)**	Standard [27,35]	<LOQ	51.81	26.48
5.69	**Methyl gallate ^b^**	C_8_H_7_O_5_^−^	183.02930	183.02985	−0.55	123.01056(35), **124.01831(100)**, 125.02362(6), **168.00384(2)**	[29]	<LOQ	<LOQ	<LOQ
6.33	**Sinapic acid ^b^**	C_11_H_11_O_5_^−^	223.06060	223.06260	−2.00	109.03047(46), 121.032(21), 123.04656(11), 125.02461(9), 135.04836(13), **137.02591(100)**, 138.03402(99), 151.04375(10), 161.02543(10), 179.03436(8)	[40]	<LOQ	<LOQ	<LOQ
2.39	**Gallic acid hexoside ^b^**	C_13_H_15_O_10_^−^	331.06650	331.06970	−3.20	124.0185(18), **125.02615(96)**, **168.00922(100)**, **169.01397(17)**, 331.07253(7)	[27]	<LOQ	27.37	34.50
6.47	**Coumaroylquinic acid isomer I ^b^**	C_16_H_17_O_8_^−^	337.09230	337.09549	−3.19	**119.05185(100)**, 120.05465(10), **163.04224(45)**, 164.0453(5), 173.04689(3), **191.05832(15)**, 192.06292(2)	[27,28]	<LOQ	-	<LOQ
7.35	**Coumaroylquinic acid isomer II ^b^**	C_16_H_17_O_8_^−^	337.09230	337.09515	−2.85	111.0467(23), **119.05188(52)**, 137.0275(11), **163.04257(28)**, **173.04812(100)**, **191.05846(7)**	[27,28]	<LOQ	-	<LOQ
2.09	**Galloylquinic acid isomer I ^b^**	C_14_H_15_O_10_^−^	343.06650	343.06970	−3.20	**125.02662(1)**, 127.04233(3), 137.02634(6), 173.04847(2), **191.05879(100)**	[27,28]	<LOQ	27.74	26.54
3.30	**Galloylquinic acid isomer II ^b^**	C_14_H_15_O_10_^−^	343.06650	343.06970	−3.20	107.01476(10), 111.04708(8), 124.01873(3), **125.02625(89)**, 137.02579(5), **169.01627(100)**, 173.0471(27), **191.05852(45)**	[27,28]	<LOQ	<LOQ	<LOQ
5.93	**Caffeoylquinic acid ^b^**	C_16_H_17_O_9_^−^	353.08730	353.09044	−3.14	134.04159(10), **135.047(97)**, 136.05015(12), 161.02425(6), 173.04671(5), **179.03619(43)**, **191.05764(100)**, 192.06487(10)	[27,41]	<LOQ	<LOQ	-
7.55	**Feruloylquinic acid ^b^**	C_17_H_19_O_9_^−^	367.10290	367.10760	−4.70	111.04851(12), 129.05678(7), 130.08874(19), **134.04015(41)**, 173.04998(24), 174.04921(9), 175.04191(7), **191.05723(100)**, 192.05995(10), **193.05302(23)**	[41]	-	<LOQ	<LOQ
6.88	**Galloyl-HHDP-hexose ^b^**	C_27_H_21_O_18_^−^	633.07280	633.07770	−4.90	169.01712(3), 249.04386(3), 275.02233(9), **301.00272(100)**, 302.00625(18), 331.07316(2), 463.05704(8), 633.0797(33)	[28,29]	26.09	<LOQ	<LOQ
**∑**								**26.09**	**198.78**	**187.83**
Flavan-3-ols and derivatives										
6.74	**Catechin ^a^**	C_15_H_13_O_6_^−^	289.07120	289.07420	−3.00	109.03155(95), 121.03162(28), 122.03861(16), **123.04722(100)**, 125.02644(43), 137.02677(28), 151.04223(32), 161.06179(13), 203.07402(20), 221.08493(13)	Standard [27]	7628.17	711.36	1283.74
7.35	**Epicatechin ^a^**	C_15_H_13_O_6_^−^	289.07120	289.07420	−3.00	109.03111(93), 121.03105(28), **123.04687(100)**, 125.02618(45), 137.02672(25), 149.02669(15), 151.04176(29), 161.06232(13), 203.07438(19), 221.08592(11)	Standard [27]	1504.70	447.58	510.25
3.91	**Gallocatechin ^c^**	C_15_H_13_O_7_^−^	305.06610	305.06920	−3.10	109.03095(15), 111.04691(22), 121.03207(5), 123.01092(12), 124.018(9), **125.02624(100)**, 137.02557(33), 139.04209(29), 165.02075(14), 167.03728(21), 219.06784(7)	[27]	709.41	783.37	581.65
4.92	**Epigallocatechin ^c^**	C_15_H_13_O_7_^−^	305.06610	305.06920	−3.10	109.03141(15), 111.04717(20), 123.01039(11), 124.01854(9), **125.02647(100)**, 137.02679(36), 139.04187(33), 165.02131(19), 167.03747(21), 219.06795(7)	[27]	1109.42	856.09	1233.48
8.83	**Epiafzelechin 3-*O*-gallate ^c^**	C_22_H_17_O_9_^−^	425.08730	425.09098	−3.68	124.01793(45), **125.02621(57)**, 137.02573(16), 149.02736(10), 168.00816(10), **169.01729(74)**, 187.08013(18), 189.05883(21), 205.08878(23), 211.07995(15), 229.08791(19), 255.07135(37), **273.07992(100)**, 274.08329(13)	[27]	107.99	-	-
8.29	**Epicatechin gallate ^c^**	C_22_H_17_O_10_^−^	441.08220	441.08710	−4.90	109.03134(5), 124.01855(11), **125.02618(45)**, **169.0168(100)**, 170.01997(8), 179.03774(4), 203.07401(6), 205.05352(5), 245.085(13), **289.07557(20)**, 290.07856(4)	[27]	2203.25	234.44	282.18
7.48	**Epigallocatechin gallate ^c^**	C_22_H_17_O_11_^−^	457.07710	457.08234	−5.24	124.01823(2), 125.02634(48), **169.01665(100)**, 170.01985(9), 305.07011(3)	[27]	829.36	138.32	158.26
4.18	**Gallocatechin hexoside ^c^**	C_21_H_23_O_12_^−^	467.11900	467.12140	−2.40	**125.02606(91)**, 137.02621(47), 165.0216(37), 167.03661(54), 179.03724(28), 219.06938(27), 261.08123(35), **305.07077(100)**, 306.07254(21), 467.12336(10)	[27]	71.56	40.61	25.07
6.40	**Epigallocatechin hexoside ^c^**	C_21_H_23_O_12_^−^	467.11900	467.12390	−4.90	**125.02594(76)**, 137.02617(51), 161.02611(20), 165.02483(39), 167.0384(86), 169.02013(19), 179.0352(51), 261.07905(32), **305.07145(100)**	[27]	73.80	-	-
7.01	**Procyanidin dimer B type ^c^**	C_30_H_25_O_12_^−^	577.13460	577.14030	−5.70	125.02592(91), 137.0266(16), 151.04409(11), 161.02718(26), 205.05107(12), 245.08291(24), 273.04275(10), **289.07448(100)**, 407.0819(73), 408.08588(28)	[27]	404.62	26.77	54.98
7.35	**Chalcan-flavan 3-ol dimer ^c^**	C_30_H_27_O_12_^−^	579.15080	579.15600	−5.20	125.02547(15), 137.02661(10), 167.03743(10), 205.05567(14), 245.08484(38), **289.07504(100)**, 290.07943(22), 369.06652(9), 459.10279(6), 579.14044(6)	[28]	-	60.44	-
6.26	**Epigallocatechin-epigallocatechin 3-*O*-gallate ^c^**	C_37_H_29_O_18_^−^	761.13540	761.14290	−7.50	125.02502(12), **169.01804(11)**, 297.04335(15), 299.05776(11), 327.056(34), 339.05854(16), 435.07646(11), 453.08687(47), 465.08847(25), 471.09785(13), 573.10382(11), **591.11999(100)**, 592.12505(36), 609.13553(70), 610.13481(32)	[27]	199.77	-	-
∑								**14,842.1**	**3298.98**	**4129.60**
Flavonol aglycones and glycosides										
10.74	**Kaempferol ^d^**	C_15_H_9_O_6_^−^	285.03990	285.04190	−2.00	143.05241(8), 151.00639(6), 159.04741(9), 171.04757(7), 185.06338(13), 187.04283(11), 211.04312(8), 229.05402(11), 239.03849(9), **285.04486(100)**, 286.04783(19)	[38]	<LOQ	1635.77	1617.70
9.91	**Quercetin ^a^**	C_15_H_9_O_7_^−^	301.03480	301.03600	−1.20	107.01558(46), 121.03135(43), 149.02665(9), **151.0061(100)**, 152.00988(10), 179.00107(13), 187.04291(3), 245.04782(4), 301.04008(4)	Standard [39]	88.55	461.76	449.41
10.85	**Isorhamnetin ^a^**	C_16_H_11_O_7_^−^	315.05050	315.05420	−3.70	107.01561(19), 151.00586(49), 152.01066(7), 227.03772(6), 243.03308(6), 255.03296(7), 271.02837(11), 283.02806(8), **300.03166(100)**, 301.03448(20)	Standard [42]	<LOQ	99.61	105.72
9.06	**Kaempferol-3-*O*-rhamnoside ^d^**	C_21_H_19_O_10_^−^	431.09780	431.10553	−7.73	227.04101(24), 255.03527(41), 256.03883(21), 257.04198(7), 271.08023(5), **284.03663(100)**, 285.04091(75), 431.10621(6)	[38]	<LOQ	-	-
8.56	**Kaempferol-3-*O*-hexoside ^d^**	C_21_H_19_O_11_^−^	447.09270	447.09790	−5.20	227.03763(21), 228.04433(3), 255.03369(32), 257.04587(3), **284.03646(100)**, 285.04159(41), 447.10039(13)	[37]	29.65	120.87	163.17
8.22	**Quercetin 3-*O*-hexoside ^d^**	C_21_H_19_O_12_^−^	463.08770	463.09320	−5.50	151.00521(4), 179.00053(3), 243.03319(2), 255.03379(6), 256.03636(1), 271.02862(11), **300.03139(100)**, 301.03764(48)	[27,38]	63.82	<LOQ	<LOQ
7.75	**Myricetin 3-*O*-hexoside ^d^**	C_21_H_19_O_13_^−^	479.08260	479.08780	−5.20	151.0069(1), 179.00116(3), 271.02806(8), 287.02337(4), **316.02649(100)**, 317.03185(29), 318.0332(5), 479.08636(4)	[27]	30.94	-	-
8.89	**Kaempferol 3-*O*-(6″-acetyl)hexoside ^d^**	C_23_H_21_O_12_^−^	489.10330	489.10871	−5.41	227.04146(5), 229.05163(5), 255.03556(11), 256.03807(5), 257.04548(7), **284.03729(100)**, 285.04252(96), 489.28126(3)	[29]	<LOQ	<LOQ	<LOQ
8.89	**Kaempferol 3-*O*-(6″-*O*-malonyl)hexoside ^d^**	C_24_H_21_O_14_^−^	533.09310	533.10034	−7.24	227.03607(2), 229.05413(2), 255.03368(7), 257.04871(2), **284.03563(94)**, **285.04255(100)**, 286.04646(18), 287.04729(2), 489.10903(5), 490.11036(2)	[26]	<LOQ	-	<LOQ
8.14	**Kaempferol 3-*O*-(2″-*O*-pentosyl)hexoside ^d^**	C_26_H_27_O_15_^−^	579.13500	579.13861	−3.61	227.03675(3), 255.03401(10), 257.04532(4), **284.03524(100)**, 285.04112(35), 429.08863(4), 579.14137(23), 580.14395(9)	[26]	-	<LOQ	<LOQ
8.36	**Kaempferol 3-*O*-(6″-*O*-rhamnosyl)hexoside ^d^**	C_27_H_29_O_15_^−^	593.15060	593.15660	−6.00	227.03779(2), 255.03327(5), 256.03779(2), 257.04991(2), **284.03638(50)**, **285.04383(100)**, 286.04755(17), 593.1586(44), 594.16158(16)	[28,38]	30.94	32.55	60.58
7.75	**Isorhamnetin 3-*O*-(6″-*O*-hexosyl)hexoside ^d^**	C_28_H_31_O_17_^−^	639.15610	639.16235	−6.25	271.02798(6), 299.02295(44), 300.02956(19), 301.03072(3), **314.04657(100)**, 315.0526(42), 459.09849(5), 639.16315(58), 640.16622(23)	[26]	<LOQ	-	<LOQ
8.35	**Kaempferol 3-*O*-(2″-pentosyl)-malonyl-pentoside ^d^**	C_29_H_31_O_17_^−^	651.15610	651.15920	−3.10	135.04763(40), 145.03209(11), 255.0313(9), **284.03597(100)**, 285.04397(45), 399.20994(52), 509.28402(13), 519.26712(52), 535.2655(8), 651.16299(40)	**/**	-	<LOQ	<LOQ
8.02	**Kaempferol 3-*O*-(2″-rhamnosyl-6″-hexosyl)hexoside ^d^**	C_33_H_39_O_20_^−^	755.20350	755.20606	−2.56	229.05162(2), 255.03363(2), 284.03595(12), **285.04393(100)**, 286.04858(17), 449.05875(4), 467.06532(5), 605.10631(2), 755.21071(84), 756.21563(38)	[26,43]	-	142.53	142.68
7.82	**Quercetin 3-*O*-(6″-rhamnosyl-hexosyl)hexoside ^d^**	C_33_H_39_O_21_^−^	771.19840	771.20540	−7.00	151.00583(1), 179.00097(1), 271.02833(1), 300.03146(16), **301.03879(24)**, **771.20752(100)**, 772.21109(44), 773.21283(13)	[27,43]	212.29	30.26	81.36
6.57	**Kaempferol 3-*O*-(6″-hexosyl)hexoside-7-*O*-hexoside ^d^**	C_33_H_39_O_21_^−^	771.19840	771.20839	−9.99	255.03458(2), 283.0282(6), **284.03554(13)**, 285.04319(10), 446.09042(9), 447.09619(4), **609.15384(100)**, 610.15678(36), 651.16692(1), 771.20778(1)	[38]	-	<LOQ	47.14
6.40	**Quercetin 3-*O*-(2″-hexosyl)hexoside-7-*O*-hexoside ^d^**	C_33_H_39_O_22_^−^	787.19330	787.20056	−7.26	178.99502(3), 299.02313(26), 300.02955(9), **301.04217(11)**, 445.08287(3), 462.08742(58), **463.09198(49)**, **625.14705(88)**, 626.15142(31), **787.20256(100)**, 788.20381(38)	[38]	-	<LOQ	<LOQ
8.02	**Quercetin 3-*O*-(6″-rhamnosyl)hexoside ^d^**	C_27_H_29_O_16_^−^	609.14569	609.15110	−5.41	151.00589(3), 179.00092(3), 255.03315(3), 271.02923(6), **300.031640(100)**, 301.03816(73)	[27,37]	164.79	155.44	193.02
7.88	**Kaempferol 3-*O*-(2″-hexosyl)hexoside ^d^**	C_27_H_29_O_16_^−^	609.14569	609.15299	−7.30	227.03677(3), 255.03261(8), **284.03562(100)**, 285.04182(49), 429.08801(5)	[38]	-	167.83	181.06
7.54	**Quercetin 3-*O*-(2″-hexosyl)hexoside ^d^**	C_27_H_29_O_17_^−^	625.14058	625.14793	−7.35	179.00092(4), 271.02771(5), **300.03166(100)**, **301.03668(30)**, 445.08254(2), 463.09269(2)	[38]	-	225.47	293.15
9.30	**Quercetin 3-*O*-(2″-rhamnosyl-3″-pentosyl-6″-coumaroyl)hexoside ^d^**	C_41_H_43_O_22_^−^	887.22465	887.23370	−9.05	151.00495(3), 179.00133(3), 299.02251(12), **300.03151(29)**, **301.03918(83)**, 723.18393(3), 741.1968(20), **887.23568(100)**	[28]	39.06	-	-
9.23	**Quercetin 3-*O*-(2″-rhamnosyl-3″-hexosyl-6″-coumaroyl)hexoside ^d^**	C_42_H_45_O_23_^−^	917.23527	917.24240	−7.13	169.01477(3), 178.99951(3), 299.01998(9), **300.03042(21)**, **301.03909(49)**, 609.14184(3), 753.19589(4), 771.20409(8), **917.24439(100)**	[28]	<LOQ	<LOQ	<LOQ
**∑**								**660.03**	**3072.07**	**3334.97**
Flavone aglycone										
10.58	**Apigenin ^a^**	C_15_H_9_O_5_^−^	269.04500	269.04763	−2.63	107.01539(26), **119.05147(20)**, 133.03093(53), 135.04631(16), **151.0058(12)**, 159.04666(22), 181.06752(15), 183.04727(15), 201.05826(11), 224.05064(12), 225.05784(9), **269.04913(100)**, 270.05167(21)	Standard	<LOQ	-	<LOQ
9.87	**Luteolin ^a^**	C_15_H_9_O_6_^−^	285.03990	285.04310	−3.20	107.01603(18), 121.03196(5), **133.03111(100)**, 134.03446(11), 151.00573(31), 175.04358(15), 199.04331(12), 217.05309(7), 285.04402(45), 286.04861(8)	Standard	364.29	-	-
**∑**								**364.29**	**0**	**0**
Flavanone aglycone										
10.58	**Naringenin ^a^**	C_15_H_11_O_5_^−^	271.06060	271.06123	−0.63	107.01562(30), **119.05205(100)**, 120.05542(11), **151.00569(31)**, 152.00874(3), 161.06313(3), 165.02226(2), 177.02197(3), 187.04301(5)	Standard	<LOQ	477.61	395.35
9.06	**Eridictyol ^e^**	C_15_H_11_O_6_^−^	287.05560	287.05723	−1.63	107.01722(11), **125.02643(100)**, 126.02753(11), 131.05415(11), 151.00542(17), 152.01339(16), 173.06236(8), 177.06071(27), 201.05888(6), 241.04914(6), 259.06193(10)	[37]	-	<LOQ	<LOQ
**∑**								**0**	**477.61**	**395.35**
**∑∑**								**15,892.5**	**7047.44**	**8047.76**

Abbreviations: Bolded fragments are the main ones for every compounds; 0—control sample (kombucha-fermented (7 days) green tea sample,); 3—sample with pollen (20 g) fermented (7 days) without kombucha; K3—beverage with pollen (20 g) fermented (7 days) with kombucha; “LOQ”—less of limit quantification; “-”—nonidentified compounds; compound quantities expressed using available standards ^a^; compounds expressed as gallic acid equivalents ^b^; compounds expressed as epicatechin equivalents ^c^; compounds expressed as quercetin equivalents ^d^; compounds expressed as naringenin equivalents ^e^. * Previously reported compounds in rapeseed/other bee pollens and/or *Camellia sinensis* tea.

**Table 3:** Identification, proposed structure/name and relative content (**%**) of phenylamides in kombucha samples with/without bee-collected pollen, using UHPLC-QToF-MS.

No	RT	Compounds (Proposed Phenylamide Name)	Formula	Calculated Mass	*m*/*z* Exact Mass	mDa	Fragments (MS^2^)	Refs. *	Samples (%)			Ratio 3/K3
									0	3	K3	
**1**	7.41	**Benzoiyl putrescin** **(*N^1^*-benzoyl putrescin)**	C_11_H_17_N_2_O^+^	193.13410	193.13470	−0.60	105.07078(9), 108.08172(12), 120.08237(11), 134.09717(8), 136.11229(27), 148.11206(13), 150.0926(43), **164.10833(100)**, 165.11111(19)	/	-	0.46	2.72	0.17
**2**	7.92	**Coumaroyl spermidine (*N^1^*-coumaroyl spermidine)**	C_16_H_26_N_3_O_2_^+^	292.20250	292.20449	−1.99	**119.05032(15)**, 146.16608(1), **147.04553(100)**, 148.0485(14), **204.10387(7)**	[38]	-	2.55	2.62	0.96
**3**	8.01	**Dicoumaroyl spermidine (*N^1^,N^5^*-dicoumaroyl spermidine)**	C_25_H_32_N_3_O_4_^+^	438.23930	438.24160	−2.30	**119.05045(8)**, 129.14018(4), **147.04577(95)**, **204.10406(100)**, 205.10683(22), 218.11927(16), 275.17809(15), **292.20439(39)**, 293.20749(10)	[26,38]	-	56.04	55.41	1.00
**4**	8.02	**Feruloyl coumaroyl spermidine (*N^1^*-feruloyl-*N^5^*-coumaroyl spermidine)**	C_26_H_34_N_3_O_5_^+^	468.24980	468.25352	−3.72	145.02926(19), **147.04528(51)**, **177.0557(100)**, 178.05894(13), **204.10298(71)**, 205.10605(12), **234.11379(63)**, 235.11715(12), 275.17737(8), 292.20353(32), 293.20609(7), 322.21375(22), 468.25352(15)	[38]	-	1.65	1.59	1.02
**5**	9.90	**Tricoumaroyl spermidine (*N^1^,N^5^,N^10^*-tri-coumaroyl spermidine)**	C_34_H_38_N_3_O_6_^+^	584.27610	584.28269	−6.59	119.05086(3), **147.0457(41)**, **204.10425(50)**, 275.1783(15), 292.20513(32), 293.20743(8), 420.23277(15), 421.22839(9), **438.24348(100)**, 439.2466(40)	[26,38]	-	0.02	0.01	1.63
**6**	8.96	**Tricoumaroyl spermine (*N^1^,N^5^,N^10^*-tri-coumaroyl spermine)**	C_37_H_45_N_4_O_6_^+^	641.33390	641.33760	−3.70	129.13967(7), **147.04515(36)**, 203.1194(14), **204.10365(84)**, 205.10674(13), 275.17735(91), 477.28941(17), **495.29994(54)**, 496.30279(23), **641.33788(100)**, 642.34076(59)	[38]	-	2.04	1.96	1.03
**7**	8.62	**Dicoumaroyl caffeoyl spermine (*N^1^,N^5^*-di-coumaroyl-*N^10^*-caffeoyl spermine)**	C_37_H_45_N_4_O_7_^+^	657.32880	657.33260	−3.80	**147.04542(22)**, **204.10404(45)**, 205.1068(7), **220.09925(17)**, 275.1777(45), 276.18055(11), 291.17267(27), 292.17816(6), 349.2620(2), 365.2564(1), **511.29547(35)**, 512.29859(15), 640.3124(2), **657.334(100)**, 658.33662(57)	[26,38]	-	4.82	4.64	1.03
**8**	8.96	**Dicoumaroyl feruloyl spermine (*N^1^,N^5^*-di-coumaroyl-*N^10^*-feruloyl spermine)**	C_38_H_47_N_4_O_7_^+^	671.34450	671.34820	−3.70	**147.04525(19)**, **177.056(11)**, 204.10379(41), 234.11506(16), 275.17771(43), 305.18806(23), 451.2254(3), 495.2997(10), 525.31078(32), 526.31495(15), **671.34824(100)**, 672.35241(61)	[26,38]	-	3.14	3.08	1.01
**9**	8.62	**Dicoumaroyl hydroxyferuloyl spermine (*N^1^,N^10^*-di-coumaroyl-*N^5^*-hydroxyferuloyl spermine)**	C_38_H_47_N_4_O_8_^+^	687.33940	687.34340	−4.00	**147.04555(16)**, **193.05083(5)**, 204.10339(39), 205.10675(6), 250.11019(14), 275.17709(39), 321.18326(23), 495.30019(11), 541.30598(32), **687.34338(100)**, 688.34674(63)	[26,38]	-	16.96	16.39	1.02
**10**	8.96	**Diferuloyl coumaroyl spermine (*N^1^,N^10^*-di-feruloyl-*N^5^*-coumaroyl spermine)**	C_39_H_49_N_4_O_8_^+^	701.35500	701.35750	−2.50	**147.0452(11)**, **177.05649(12)**, 204.10372(30), 234.1151(9), 275.17743(34), 276.18067(7), 305.18736(14), 335.20036(9), 525.30953(13), 555.3214(18), **701.35917(100)**, 702.36096(65), 703.35572(30)	[38]	-	1.37	1.21	1.12
**11**	8.36	**Coumaroyl hydroxyferuloyl caffeoyl spermine (*N^1^*-coumaroyl-*N^5^*-hydroxyferuloyl-*N^10^*-caffeoyl spermine)**	C_38_H_47_N_4_O_9_^+^	703.33430	703.33870	−4.40	**147.0451(7)**, 204.1034(3), **220.0986(24)**, **250.1098(11)**, 275.1770(4), 291.1721(26), 321.1830(17), 467.2221(2), 511.2947(8), 541.3054(11), 557.3008(17), 686.3193(1), **703.3387 (100)**, 704.3422(64)	[26,38]	-	4.38	4.32	1.00
**12**	8.69	**Coumaroyl hydroxyferuloyl feruloyl spermine (*N^1^*-coumaroyl-*N^5^*-hydroxyferuloyl-*N^10^*-feruloyl spermine)**	C_39_H_49_N_4_O_9_^+^	717.35000	717.35390	−3.90	**147.04485(5)**, **177.05619(10)**, 204.10388(10), 234.11428(15), **250.11079(9)**, 275.17762(13), 305.18827(15), 321.1835(15), 525.31035(7), 541.30497(11), 553.30564(5), 571.31718(12), **717.35426(100)**, 718.35733(62)	[26,38]	-	6.10	5.64	1.07
**13**	9.57	**Tricaffeoyl hydroxyferuloyl spermine**	C_47_H_53_N_4_O_13_^+^	881.36090	881.36498	−4.08	**220.09787(4)**, **250.10682(3)**, 291.17122(6), 321.18306(4), 557.29922(7), 689.32273(4), 701.32262(5), **719.33302(100)**, 720.33609(64), 881.36498(9)	[26,38]	-	0.47	0.41	1.14
	∑Total area 3/∑Total area K3											0.99

Abbreviations: Bolded fragments are the main ones for every compounds; 0—control sample (kombucha-fermented (7 days) green tea sample); 3—sample with pollen (20 g) fermented (7 days) without kombucha; K3—beverage with pollen (20 g) fermented (7 days) with kombucha; “-”—nonidentified compounds; The relative content (%) of individual phenylamide in each sample is given. Target compounds, expected retention time (RT), molecular formula, calculated mass, exact mass, and MS^2^ fragments are presented. * Previously reported compounds in rapeseed or other bee pollens.

**Table 4:** Antimicrobial activity of kombucha samples enriched with BCP expressed as MIC, MBC, and MFC.

Sample		*P. aeruginosa*	S. Enteritidis	*K. aerogenes*	*A. baumannii*	*E. coli*	*S. aureus* ATCC	*S. aureus* Clinical Isolate	*L. monocytogenes*	*B. spizizenii*	*C. albicans*
Antibiotic/Antimycotic * (mg/mL)	MIC	2.5 ± 0.0	<0.05	1.7 ± 0.7	1.6 ± 0.0	<0.05	<0.05	<0.05	<0.05	<0.05	0.3 ± 0.0
	MBC	13.3 ± 5.7	0.05 ± 0.0	6.7 ± 2.9	2.5 ± 0.0	0.05 ± 0.0	<0.05	<0.05	<0.05	<0.05	0.3 ± 0.0
0	MIC	50.0 ± 0.0% ^a^	-	6.3 ± 0.0% ^d^	-	-	-	50.0 ± 0.0% ^a^	-	-	-
	MBC/ MFC	-	-	-	-	-	-	-	-	-	-
0N	MIC	-	-	-	-	-	-	50.0 ± 0.0% ^a^	-	-	-
	MBC/ MFC	-	-	-	-	-	-	-	-	-	-
1	MIC	6.3 ± 0.0% ^d^	1.6 ± 0.0% ^e^	6.3 ± 0.0% ^d^	6.3 ± 0.0% ^d^	3.1 ± 0.0% ^c^	3.1 ± 0.0% ^e^	3.1 ± 0.0% ^e^	12.5 ± 0.0% ^c^	25.0 ± 0.0% ^b^	50.0 ± 0.0% ^a^
	MBC/ MFC	12.5 ± 0.0% ^c^	25.0 ± 0.0% ^a^	25.0 ± 0.0% ^b^	12.5 ± 0.0% ^c^	12.5 ± 0.0% ^a^	25.0 ± 0.0% ^b^	25.0 ± 0.0% ^b^	50.0 ± 0.0% ^a^	25.0 ± 0.0% ^b^	50.0 ± 0.0% ^a^
1N	MIC	50.0 ± 0.0% ^a^	3.1 ± 0.0% ^d^	12.5 ± 0.0% ^c^	50.0 ± 0.0% ^a^	6.3 ± 0.0% ^b^	-	-	25.0 ± 0.0% ^b^	-	-
	MBC/ MFC	-	-	-	-	-	-	-	-	-	-
K1	MIC	3.1 ± 0.0% ^e^	1.6 ± 0.0% ^e^	6.3 ± 0.0% ^d^	6.3 ± 0.0% ^d^	3.1 ± 0.0% ^c^	12.5 ± 0.0% ^c^	12.5 ± 0.0% ^c^	6.3 ± 0.0% ^d^	12.5 ± 0.0% ^c^	50.0 ± 0.0% ^a^
	MBC/ MFC	6.3 ± 0.0% ^d^	6.3 ± 0.0% ^c^	25.0 ± 0.0% ^b^	12.5 ± 0.0% ^c^	12.5 ± 0.0% ^a^	50.0 ± 0.0% ^a^	12.5 ± 0.0% ^c^	25.0 ± 0.0% ^b^	12.5 ± 0.0% ^c^	-
K1N	MIC	50.0 ± 0.0% ^a^	3.1 ± 0.0% ^d^	-	50.0 ± 0.0% ^a^	12.5 ± 0.0% ^a^	-	-	25.0 ± 0.0% ^b^	-	-
	MBC/ MFC	-	-	-	-	-	-	-	-	-	-
2	MIC	3.1 ± 0.0% ^e^	1.6 ± 0.0% ^e^	6.3 ± 0.0% ^d^	6.3 ± 0.0% ^d^	3.1 ± 0.0% ^c^	12.5 ± 0.0% ^c^	1.6 ± 0.0% ^f^	6.3 ± 0.0% ^d^	12.5 ± 0.0% ^c^	25.0 ± 0.0% ^b^
	MBC/ MFC	6.3 ± 0.0% ^d^	6.3 ± 0.0% ^c^	12.5 ± 0.0% ^c^	6.3 ± 0.0% ^d^	6.3 ± 0.0% ^b^	50.0 ± 0.0% ^a^	25.0 ± 0.0% ^b^	25.0 ± 0.0% ^b^	12.5 ± 0.0% ^c^	50.0 ± 0.0% ^a^
2N	MIC	50.0 ± 0.0% ^a^	3.1 ± 0.0% ^d^	12.5 ± 0.0% ^c^	50.0 ± 0.0% ^a^	6.3 ± 0.0% ^b^	-	-	25.0 ± 0.0% ^b^	-	-
	MBC/ MFC	-	-	-	-	-	-	-	-	-	-
K2	MIC	3.1 ± 0.0% ^e^	1.6 ± 0.0% ^e^	6.3 ± 0.0% ^d^	3.1 ± 0.0% ^e^	3.1 ± 0.0% ^c^	12.5 ± 0.0% ^c^	6.3 ± 0.0% ^d^	6.3 ± 0.0% ^d^	12.5 ± 0.0% ^c^	25.0 ± 0.0% ^b^
	MBC/ MFC	6.3 ± 0.0% ^d^	12.5 ± 0.0% ^b^	12.5 ± 0.0% ^c^	6.3 ± 0.0% ^d^	6.3 ± 0.0% ^b^	50.0 ± 0.0% ^a^	6.3 ± 0.0% ^d^	25.0 ± 0.0% ^b^	12.5 ± 0.0% ^c^	25.0 ± 0.0% ^b^
K2N	MIC	50.0 ± 0.0% ^a^	3.1 ± 0.0% ^d^	12.5 ± 0.0% ^c^	25.0% ^b^	12.5 ± 0.0% ^a^	50.0 ± 0.0% ^a^	50.0 ± 0.0% ^a^	25.0 ± 0.0% ^b^	-	-
	MBC/ MFC	-	-	-	-	-	-	-	-	-	-
3	MIC	3.1 ± 0.0% ^d^	1.6 ± 0.0% ^e^	3.1 ± 0.0% ^e^	3.1% ^e^	3.1 ± 0.0% ^c^	6.3 ± 0.0% ^d^	1.6 ± 0.0% ^f^	6.3 ± 0.0% ^d^	12.5 ± 0.0% ^c^	25.0 ± 0.0% ^b^
	MBC/ MFC	3.1 ± 0.0% ^e^	12.5 ± 0.0% ^b^	12.5 ± 0.0% ^c^	6.3% ^d^	6.3 ± 0.0% ^b^	25.0 ± 0.0% ^b^	12.5 ± 0.0% ^c^	25.0 ± 0.0% ^b^	12.5 ± 0.0% ^c^	25.0 ± 0.0% ^b^
3N	MIC	12.5 ± 0.0% ^c^	3.1 ± 0.0% ^d^	12.5 ± 0.0% ^c^	25.0% ^b^	6.3 ± 0.0% ^b^	25.0 ± 0.0% ^b^	50.0 ± 0.0% ^a^	25.0 ± 0.0% ^b^	-	-
	MBC/ MFC	-	-.	-	-	-	-	-	-	-	-
K3	MIC	3.1 ± 0.0% ^e^	1.6 ± 0.0% ^e^	3.1 ± 0.0% ^e^	3.1% ^e^	3.1 ± 0.0% ^c^	6.3 ± 0.0% ^d^	1.6 ± 0.0% ^f^	6.3 ± 0.0% ^d^	6.3 ± 0.0% ^d^	25.0 ± 0.0% ^b^
	MBC/ MFC	6.3 ± 0.0% ^d^	12.5 ± 0.0% ^b^	12.5 ± 0.0% ^c^	6.3% ^d^	6.3 ± 0.0% ^b^	50.0 ± 0.0% ^a^	6.3 ± 0.0% ^d^	12.5 ± 0.0% ^c^	12.5 ± 0.0% ^c^	25.0 ± 0.0% ^b^
K3N	MIC	12.5 ± 0.0% ^c^	3.1 ± 0.0% ^d^	12.5 ± 0.0% ^c^	25% ^b^	12.5 ± 0.0% ^a^	50.0 ± 0.0% ^a^	50.0 ± 0.0% ^a^	25.0 ± 0.0% ^b^	50.0 ± 0.0% ^a^	-
	MBC/ MFC	25.0 ± 0.0% ^b^	-	50.0 ± 0.0% ^a^	-	-	-	-	-	-	-

Different superscript letters in the same column indicate statistically significant differences (*p* < 0.05) among samples; * Penicillin was used to test the susceptibility of bacteria to antibiotics. Nystatin was used to test the susceptibility of yeast.

## Data Availability

Data will be made available on request.

## Supplementary Materials

The following supporting information can be downloaded at: https://www.mdpi.com/article/10.3390/antiox14060752/s1, Table S1: Equation parameters of phenolic standards used for quantification.
