# Supplementary material for: Influence of Kombucha Fermentation on Antioxidant and Antimicrobial Activity of Monofloral Rapeseed Bee-Collected Pollen
Source: Antioxidants (Basel). 2025 Jun 18;14(6):752. doi: 10.3390/antiox14060752 (PMC12189435; doi:10.3390/antiox14060752)
Supplement: Supplementary file 1 [file antioxidants-14-00752-s001.zip › antioxidants-3617610-supplementary.pdf]

Table S1. Equation parameters of used phenolic standards for quantification.

Standards	Y = a*x ± b	R^2	LOQ (ppm)
Gallic acid	y= 4611996.1798 * x - 637903.8099	0.9964	2.58
Catechin	y= 120612.0120 * x + 43508.9771	0.9924	2.04
Epicatechin	y= 212084.9086 * x + 42453.8207	0.9907	1.93
Quercetin	y= 769214.5670 * x + 825343.6827	0.9915	2.64
Isorhamnetin	y= 1084944.2566 * x + 1008067.9239	0.9936	2.82
Apigenin	y= 215501.5584 * x + 64892.2142	0.9942	2.93
Luteolin	y= 108947.9628 * x + 80537.0356	0.9958	2.51
Naringenin	y= 520364.5388 * x + 102836.6503	0.9867	4.49
